# Functional Outcomes Following Amputation in Electrical Burn Injuries: A Prospective Observational Study Utilizing the Sickness Impact Profile (SIP) and International Classification of Impairments, Disabilities, and Handicaps (ICIDH) Frameworks

**DOI:** 10.7759/cureus.108334

**Published:** 2026-05-06

**Authors:** Akshay Sankhla, Avdhesh K Sharma, Vijay Verma

**Affiliations:** 1 General Surgery, Dr. Sampurnanand Medical College, Jodhpur, IND

**Keywords:** disability evaluation, electrical burn, functional outcome, icidh, rehabilitation, sickness impact profile

## Abstract

Background and aim

Electrical burns represent a severe injury subtype with disproportionately high rates of limb amputation. While amputation may be life-preserving, its impact on long-term function remains inadequately characterized in low-resource settings. This study aimed to prospectively evaluate functional outcomes following amputation in electrical burn patients using validated instruments and identify modifiable predictors of disability.

Methods

A prospective observational study was conducted from July 2024 to July 2025 at a tertiary burn center. Forty-nine consecutive patients (>12 years) undergoing amputation for electrical burns were enrolled. Exclusion criteria included crush injury mechanisms, preexisting neurovascular comorbidities, chronic kidney disease, and mortality during treatment. The primary outcome was functional status at three months postamputation, assessed using the validated Sickness Impact Profile (SIP) and a content-validated International Classification of Impairments, Disabilities, and Handicaps-based questionnaire developed from the International Classification of Functioning, Disability and Health core sets for upper limb amputation. Statistical analysis employed IBM SPSS Statistics for Windows, version 26.0 (released 2018; IBM Corp., Armonk, NY, USA).

Results

The cohort comprised 35 males (71.4%) and 14 females (28.6%), with a mean age of 29.2 ± 10.7 years. High-voltage injuries (>1000 V) accounted for 41 cases (83.7%). Amputation levels included below elbow in 26 patients (53.1%), bilateral/above elbow in 12 patients (24.5%), and digit disarticulations in eight patients (16.3%). SIP scores demonstrated a gradient inversely related to preserved limb length: bilateral/above elbow (36.8 ± 6.5), below elbow (23.1 ± 4.8), and digit disarticulations (17.3 ± 1.9; p < 0.001). High-voltage injuries correlated with significantly higher SIP scores vs. low-voltage injuries (26.8 ± 6.5 vs. 17.3 ± 1.9, p < 0.001). Multivariate analysis identified preoperative anemia (hemoglobin <10 g/dL; β = +3.1, p = 0.047) and poor patient motivation (β = +4.3, p = 0.031) as independent predictors of worse functional outcomes.

Conclusions

Amputation level and injury voltage are primary determinants of postamputation disability. Preoperative anemia and psychological state were associated with worse functional outcomes, suggesting they may be targets for preoperative optimization pending interventional validation. These findings suggest that integrated care pathways incorporating preoperative optimization warrant prospective evaluation and structured psychosocial rehabilitation. Limitations include a modest sample size (n = 49) from a single center, which may limit generalizability and precision of subgroup estimates.

## Introduction

Electrical injuries constitute a devastating subset of burn trauma, accounting for 5-20% of burn unit admissions in developing nations. These injuries are characterized by deep tissue destruction following current passage, with high-voltage exposures (>1000 V) frequently necessitating limb amputation. Reported amputation rates approach 68% in high-voltage electrical injuries, imposing substantial long-term disability [1-3].

While the acute surgical management of electrical burns is well established, functional outcomes following amputation remain insufficiently characterized, particularly in resource-limited settings where rehabilitation services are constrained [4]. Existing literature predominantly comprises retrospective series with limited standardized outcome measurement. Specifically, no prospective study has simultaneously quantified postamputation disability using both the Sickness Impact Profile (SIP) and International Classification of Impairments, Disabilities, and Handicaps (ICIDH) frameworks in a low-resource setting. Furthermore, modifiable predictors of poor functional recovery, such as preoperative anemia and patient motivation, have not been systematically examined in electrical burn amputees. To address these gaps, this prospective study aimed to: first, quantify functional outcomes following amputation for electrical burns using SIP and ICIDH instruments; second, characterize demographic and injury patterns in this population; and third, identify modifiable predictors of poor functional recovery to inform targeted interventions. A comprehensive understanding of functional determinants is crucial for prognostication, rehabilitation planning, and healthcare resource allocation [5].

The SIP provides validated measurement of health-related dysfunction across physical and psychosocial domains [5], while the ICIDH/International Classification of Functioning, Disability and Health (ICF) framework facilitates structured assessment of activity limitations [6,7]. Combined application of these instruments offers a multidimensional evaluation of postamputation function.

The study by Greive and Lankhorst [8] demonstrated the utility of prospective functional assessment in amputee populations, while Dillingham et al. [9] provided an epidemiological context for limb loss that informs rehabilitation planning. Although these studies focused on general amputee populations, their findings regarding functional assessment methodology and prognostic factors are directly applicable to electrical burn-related amputations, as the latter represent a subset of major traumatic limb loss with similarly profound disability profiles [10]. While retrospective series have described amputation rates and injury patterns [11,12], no prospective study to date has simultaneously quantified postamputation disability using both SIP and ICF frameworks in a low-resource setting nor systematically examined factors such as preoperative anemia and patient motivation.

This prospective study aimed to quantify functional outcomes following amputation for electrical burns using SIP and ICIDH instruments; characterize demographic and injury patterns in this population; and identify factors potentially amenable to preoperative optimization that merit further investigation as predictors of poor functional recovery to inform targeted interventions.

## Materials and methods

Study design and setting

This prospective observational study was conducted in the Department of General Surgery at Mahatma Gandhi Hospital, Jodhpur, a tertiary care facility serving western Rajasthan. The study protocol received approval from the Institutional Ethics Committee (SNMC/IEC/2025/Plan/1035) and was conducted in accordance with the Declaration of Helsinki principles. Written informed consent was obtained from all participants.

Participants

Consecutive patients presenting between July 2024 and July 2025 with electrical burns requiring amputation were screened for eligibility. For the purpose of this study, “major amputation” was defined as amputation through or proximal to the wrist (including below elbow, above elbow, and bilateral upper limb amputations), while “minor amputation” was defined as amputation distal to the wrist (including digit disarticulations and partial hand amputations). Detailed inclusion and exclusion criteria are presented in Table 1.

**Table 1:** Inclusion and exclusion criteria

Criterion type	Details
Inclusion criteria	Age greater than 12 years
	Electric burn injury
	Undergoing major and minor amputations during hospitalization
Exclusion criteria	Amputation due to crush injury
	Preexisting peripheral neuropathy, vascular disease, or coagulopathy
	Chronic kidney disease (stage 3b or higher)
	Mortality due to acute hospitalization

Sample size calculation

Sample size was determined using the formula for estimating a population mean:

$n = \left(\frac{Z_{1-\alpha/2} \cdot \sigma}{E}\right)^2,$

with Z = 1.96 (95% confidence) and σ = 18 (expected SD from prior literature [9]). Determination was guided by the extent of nonviable tissue, with preservation of functional length prioritized when feasible. Postoperative care included analgesia, antibiotic prophylaxis, and stump management.

Outcome measures

The primary outcome was functional status at three months postamputation, assessed using two instruments. First, the SIP: a 136-item questionnaire yielding a percentage score (0-100%), with higher scores indicating greater dysfunction. The physical dimension subscore (including body care, ambulation, and mobility) was utilized as the primary continuous outcome measure. Second, an ICIDH-based assessment, a structured questionnaire evaluating seven functional domains (body position transfers, maintaining position, transferring self, lifting/carrying, fine hand use, hand/arm use, and moving around) on a four-point ordinal scale (0 = no problem to 3 = severe problem). While the ICIDH-based questionnaire has not undergone formal psychometric validation, its content validity is supported by direct operationalization of ICIDH activity codes (d410-d455) and expert consensus during domain selection, consistent with published ICIDH-linked outcome methodology. Motivation was assessed using the “energy and drive functions” domain (b130) of the ICIDH-based questionnaire. Patients rated items on a 0 to 3 scale (0 = no problem; 3 = severe problem). A domain score ≥2 was classified as “poor motivation” for multivariate analysis. While this approach lacks formal psychometric validation, it is derived directly from the ICIDH framework, which ensures content validity. Secondary outcomes included demographic patterns, injury characteristics, and identification of risk factors for poor functional recovery. The three-month follow-up was selected as the primary endpoint because this period captures early functional recovery, when most modifiable predictors (e.g., anemia or motivation) exert their greatest influence, and because longer follow-up in our rural, low-resource setting would risk prohibitive attrition.

Data collection

Baseline demographic, injury, and treatment data were prospectively recorded. The ICIDH assessment was performed at admission (establishing a pre-injury baseline) and at a three-month follow-up. Motivation was evaluated through the ICIDH domain “energy and drive functions (b130),” which includes items on initiative, goal-directed behavior, and engagement in daily activities. Responses were given on a 0 to 3 scale (0 = no problem, 3 = severe problem). For regression analysis, scores ≥2 were defined as poor motivation. The SIP questionnaire was administered at the three-month follow-up through interviewer-assisted completion. Follow-up assessments were conducted in person during clinic visits or via structured telephone interviews when in-person attendance was not feasible. Although telephone administration of the SIP has been reported to yield comparable results to in-person administration in disabled populations, we acknowledge the potential for mode-related measurement bias and have therefore limited telephone interviews to patients with genuine access barriers (n = 8 out of 49).

Statistical analysis

Data were analyzed using IBM SPSS Statistics for Windows, version 26.0 (released 2018; IBM Corp., Armonk, NY, USA). Continuous variables were expressed as mean ± SD or median with IQR based on distribution normality assessed by the Shapiro-Wilk test. Categorical variables were presented as frequencies and percentages. Between-group comparisons utilized Student’s t-test or Mann-Whitney U test for continuous variables and chi-square or Fisher’s exact test for categorical variables. Correlation analysis employed Pearson’s or Spearman’s coefficients, as appropriate. Multivariate linear regression identified independent predictors of SIP scores. Linear regression assumptions were verified: normality of residuals (Shapiro-Wilk test, p = 0.61), absence of multicollinearity (all variance inflation factors <2), and homoscedasticity (Breusch-Pagan test, p = 0.34). All tests were two-tailed, with statistical significance set at p < 0.05.

## Results

Participant characteristics

Forty-nine patients meeting the inclusion criteria completed the three-month follow-up. Demographic and clinical characteristics are summarized in Table 2. The cohort was predominantly male, with 35 patients (71.4%), and had a mean age of 29.2 ± 10.7 years. Most injuries occurred in occupational settings, accounting for 33 cases (67.3%), primarily involving electrical utility work in 19 patients (38.8%) and agriculture in 14 patients (28.6%). High-voltage injuries (>1000 V) accounted for 41 cases (83.7%). Baseline characteristics are presented for the entire cohort; comparisons across amputation severity groups are not shown here, as potential confounding factors were addressed in the multivariate regression model.

**Table 2:** Baseline characteristics of study participants (n = 49)

Characteristic	N (%) or mean ± SD
Demographics	
Age (years)	29.2 ± 10.7
Male gender	35 (71.4%)
Rural residence	42 (85.7%)
Injury characteristics	41 (83.7%)
Occupational injury	33 (67.3%)
Entry point: upper limb	47 (95.9%)
Associated inhalation injury	3 (6.1%)
Total body surface area burned	18.3 ± 9.7%
Amputation details	
Below elbow	26 (53.1%)
Above elbow	9 (18.4%)
Bilateral upper limb	3 (6.1%)
Digit disarticulation	8 (16.3%)
Other (shoulder or wrist)	3 (6.1%)
Comorbidities	
Any comorbidity	21 (42.9%)
Anemia (hemoglobin <10 g/dL)	7 (14.3%)
Diabetes mellitus	5 (10.2%)
Current smoking	11 (22.4%)

SIP score analysis

The mean SIP physical dimension score at three months was 25.8 ± 7.2%. As presented in Table 3, SIP scores demonstrated significant variation by amputation level, with bilateral/above-elbow amputations associated with the highest disability scores (36.8 ± 6.5). High-voltage injuries were associated with significantly higher SIP scores compared to low-voltage injuries (mean difference 9.5, 95% CI: 4.8-14.2; 26.8 ± 6.5 vs. 17.3 ± 1.9; p < 0.001). Patients with preoperative anemia demonstrated worse functional outcomes (mean difference 3.1, 95% CI: 0.05-6.15; 28.1 ± 4.2 vs. 25.0 ± 7.3; p = 0.047).

**Table 3:** SIP scores stratified by amputation level and injury characteristics SIP, Sickness Impact Profile

Variables	Category	N	Mean SIP score ± SD	p-value
Amputation levels	Bilateral/above elbow	12	36.8 ± 6.5	<0.001
	Below elbow	26	23.1 ± 4.8	
	Digit disarticulation	8	17.3 ± 1.9	
Voltage	High (>1000V)	41	26.8 ± 6.5	<0.001
	Low (≤1000V)	8	17.3 ± 1.9	
Pre-op anemia	Present (hemoglobin <10)	7	28.1 ± 4.2	0.047
	Absent	42	25.0 ± 7.3	
Age group	<30 years	31	26.2 ± 6.8	0.51
	≥30 years	18	25.1 ± 7.9	

**Figure 1:**
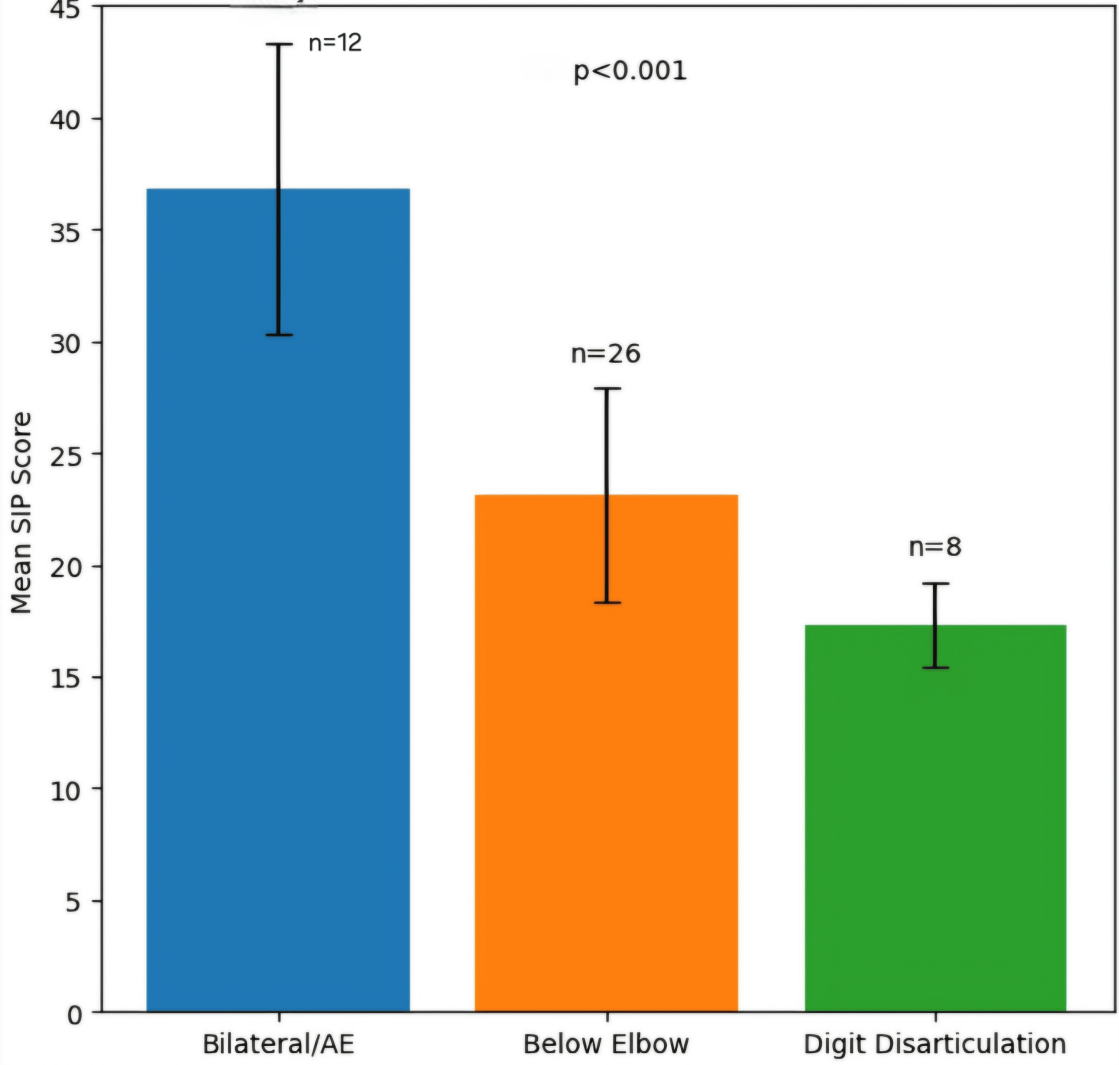
SIP score by amputation level AE, above elbow; SIP, Sickness Impact Profile

**Figure 2:**
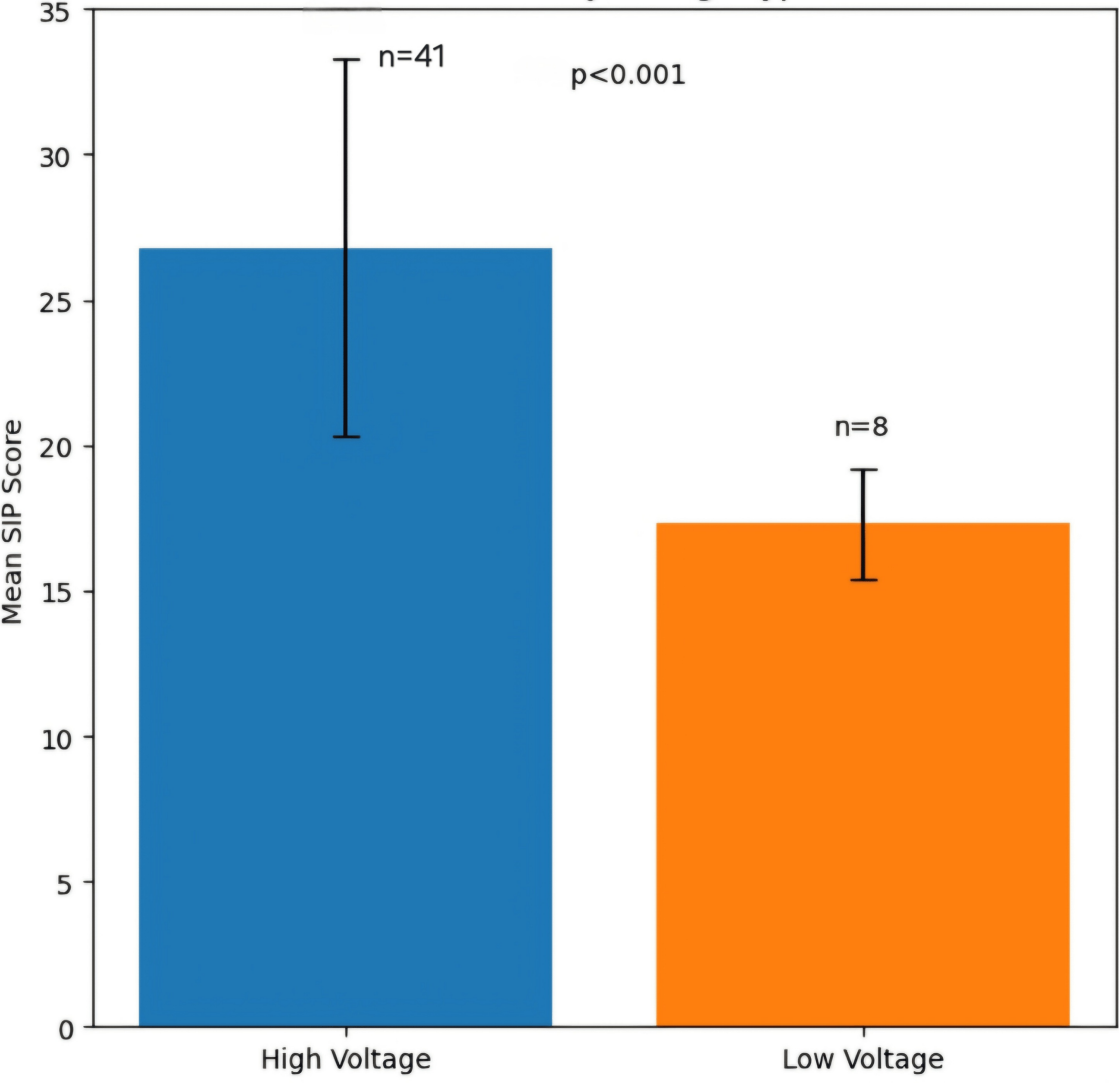
SIP scores by voltage type SIP, Sickness Impact Profile

The relationship between amputation level and SIP scores is illustrated in Figure 1. The three bars represent: bilateral/above elbow (36.8 ± 6.5, n = 12), below elbow (23.1 ± 4.8, n = 26), and digit disarticulation (17.3 ± 1.9, n = 8), which shows a clear gradient of increasing disability with more proximal amputation levels (χ² for trend = 15.3, df = 2, p < 0.001, Cramér’s V = 0.56). Similarly, Figure 2 shows two bars representing high voltage (26.8 ± 6.5, n = 41) and low voltage (17.3 ± 1.9, n = 8), demonstrating a significant difference in SIP scores between high- and low-voltage injuries (t = 4.85, df = 47, p < 0.001, Cohen’s d = 1.42).

ICIDH domain assessment

**Figure 3:**
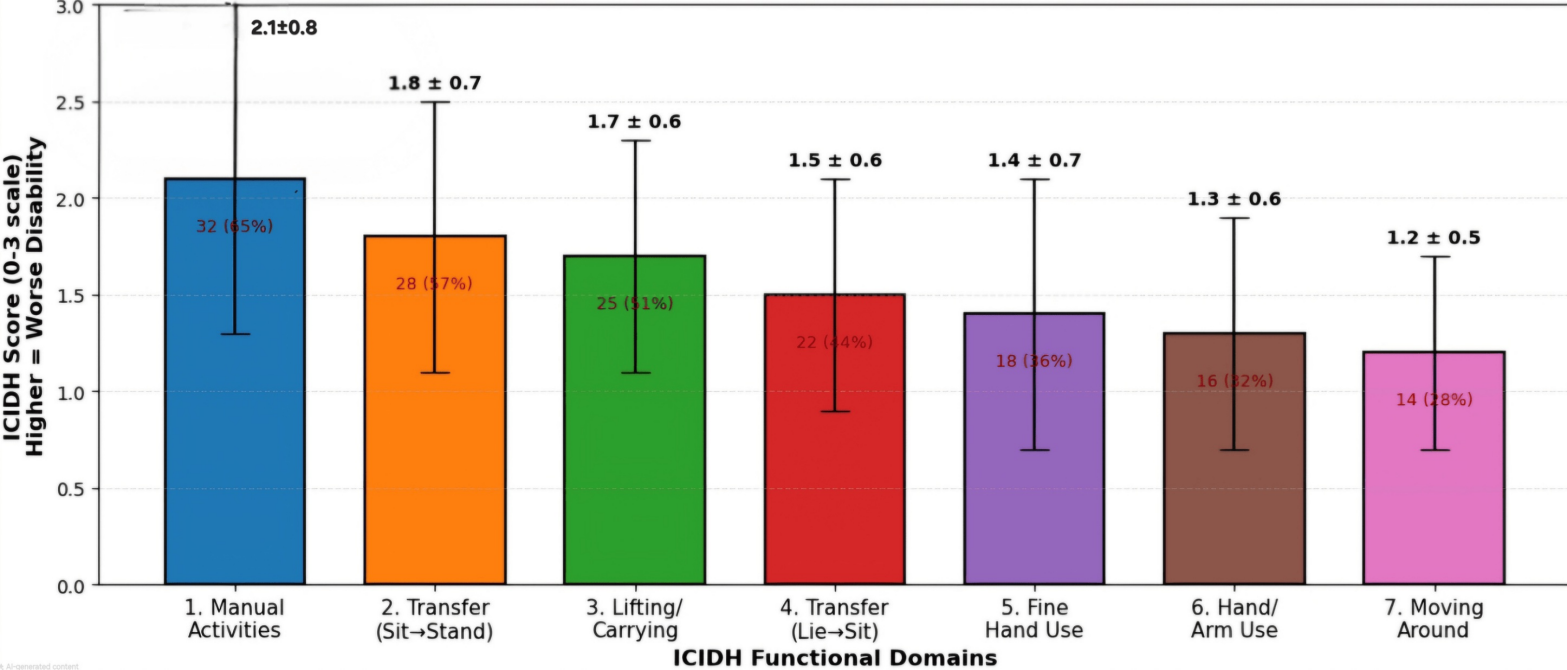
ICIDH domain-specific disability scores at three months postamputation ICIDH, International Classification of Impairments, Disabilities, and Handicaps

ICIDH-based evaluation revealed domain-specific limitations, as shown in Figure 3. The most severely affected domains were manual activities (mean score 2.1 ± 0.8), transfers from sitting to standing (1.8 ± 0.7), and lifting/carrying objects (1.7 ± 0.6). Patients undergoing subclavian vessel ligation, indicating proximal vascular injury, showed particular impairment in transfer activities. Table 4 provides a detailed breakdown of the ICIDH domain scores.

**Table 4:** ICIDH domain scores at three months postamputation ICIDH, International Classification of Impairments, Disabilities, and Handicaps

ICIDH domain	Mean score ± SD (0-3 scale)	Patients with severe disability (score ≥2), n (%)
Manual activities	2.1 ± 0.8	32 (65.3%)
Transfer (sitting to standing)	1.8 ± 0.7	28 (57.1%)
Lifting/carrying objects	1.7 ± 0.6	25 (51.0%)
Transfer (lying to sitting)	1.5 ± 0.6	22 (44.9%)
Fine hand use	1.4 ± 0.7	18 (36.7%)
Hand and arm use	1.3 ± 0.6	16 (32.7%)
Moving around	1.2 ± 0.5	14 (28.6%)

Predictors of functional outcome

Multivariate linear regression identified independent predictors of higher SIP scores, as detailed in Table 5. Bilateral/above-elbow amputation (β = +13.7, 95% CI: 9.2-18.2, p < 0.001), high-voltage injury (β = +8.9, 95% CI: 3.4-14.4, p = 0.002), preoperative anemia (β = +3.1, 95% CI: 0.05-6.15, p = 0.047), and poor patient motivation (β = +4.3, 95% CI: 0.4-8.2, p = 0.031) were associated with worse functional outcomes. Age, gender, and smoking status did not demonstrate independent predictive value. All linear regression assumptions were satisfied.

**Table 5:** Multivariate linear regression for predictors of SIP score Adjusted R² = 0.64 SIP, Sickness Impact Profile

Predictor	β coefficient	95% CI	p-value
Bilateral/above-elbow amputation	13.7	9.2 to 18.2	<0.001
High-voltage injury	8.9	3.4 to 14.4	0.002
Preoperative anemia	3.1	0.05 to 6.15	0.047
Poor motivation	4.3	0.4 to 8.2	0.031
Age (per decade)	0.8	-0.5 to 2.1	0.22
Male gender	-1.2	-4.1 to 1.7	0.41

## Discussion

This prospective study quantifies the substantial functional burden following amputation for electrical burns, identifying both injury-related and modifiable determinants of recovery. By comparing our results with existing literature, we can better understand the unique contributions and clinical implications of this research.

Amputation level and functional outcomes

The strong correlation between amputation level and SIP scores aligns with established literature. The gradient we demonstrated, with bilateral/above-elbow amputations showing mean SIP scores of 36.8 ± 6.5, nearly double those of below-elbow amputations at 23.1 ± 4.8, provides quantitative confirmation of the exponential functional impact of proximal limb loss. This finding is consistent with Dillingham et al., who reported that more proximal amputations are associated with significantly greater disability [9], and with Greive and Lankhorst, who demonstrated that functional outcome is inversely related to amputation level [8]. The particularly high disability scores for bilateral and above-elbow amputations underscore the need for intensive, targeted rehabilitation resources for this subgroup, as advocated by Sinha et al. [10].

High-voltage injuries and occupational patterns

The predominance of high-voltage injuries (83.7%) in our cohort reflects occupational exposure patterns in western Rajasthan and is consistent with reports from other developing economies. Dash et al. reported similar findings, with 78% of their Indian cohort sustaining high-voltage injuries in occupational settings [11], while Hsueh et al. identified work-related activities as the primary mechanism and injury voltage as the strongest predictor of limb amputation [12]. The significantly worse outcomes associated with high-voltage exposure in our study (SIP scores 26.8 ± 6.5 vs. 17.3 ± 1.9; p < 0.001) align with Arnoldo et al., who documented that high-voltage injuries result in more extensive tissue destruction and prolonged rehabilitation compared to low-voltage injuries [1].

Preoperative anemia as a modifiable risk factor

Preoperative anemia (hemoglobin < 10 g/dL) emerged as an independent predictor of worse functional outcomes (β = +3.1, p = 0.047). Shander et al. demonstrated that preoperative anemia is associated with increased morbidity across surgical specialties and advocated for routine hemoglobin optimization [13]. The mechanism likely involves reduced oxygen delivery to healing tissues, supported by Pell and Stonebridge who identified low hemoglobin as a predictor of poor functional outcomes in trauma amputees [14]. Whether preoperative anemia correction directly improves functional outcomes requires confirmation in an interventional trial. Because this study is observational, all reported associations (including those for anemia and motivation) should be interpreted as correlational, not causal.

Patient motivation and psychological factors

The significant association between patient motivation and functional recovery (β = +4.3, p = 0.031) reinforces the importance of psychological factors. Wiechman et al. documented that depression and anxiety persist beyond the acute injury phase and are associated with poorer functional outcomes [15]. Patterson et al. described the complex interplay between physical recovery and mental health, noting that positive psychological adjustment improves rehabilitation adherence [16]. Esselman et al. called for integrated care models addressing both physical and psychological needs from acute care through community reintegration [17]. Our findings provide quantitative confirmation that poor motivation (as assessed by the ICIDH energy/drive domain) independently predicts higher disability scores. However, we did not assess other psychological constructs such as depression, anxiety, or coping styles; therefore, our data do not directly support broader psychological interventions beyond addressing motivation.

Age and gender considerations

Our finding that age was not predictive of outcomes contrasts with some studies. Ebrahimzadeh and Fattahi found that older age was associated with poorer functional outcomes in amputees [18], and Pell and Stonebridge demonstrated that age independently predicted worse outcomes after traumatic amputation [14]. The discrepancy may reflect the younger demographic in our cohort (mean age 29.2 ± 10.7 years), consistent with Bartley et al., who noted that age becomes more important in older patients with comorbidities [5]. The lack of gender-based difference aligns with Dillingham et al., who found no significant gender differences after controlling for amputation level and etiology [9].

Our findings diverge from some published reports. Sullivan et al. [19] found that age >40 years independently predicted poor functional outcomes after electrical burn injury, which we did not observe. This discrepancy likely reflects our cohort’s younger mean age (29.2 vs. 38.5 years) and lower prevalence of age-related comorbidities. We found no gender-based difference, which may be attributable to our predominantly male, occupational injury sample, where amputation levels and injury voltage were comparable between genders. This may be attributable to our predominantly male, occupational injury sample, where amputation levels and injury voltage were comparable between genders. These divergent findings underscore the importance of population-specific prognostic factors and caution against generalizing results across age and gender distributions.

Clinical implications

Synthesizing our findings with existing literature suggests actionable strategies: preoperative anemia correction as advocated by Shander et al. [13], intensive rehabilitation for proximal amputations as supported by Greive and Lankhorst [8] and Sinha et al. [10], psychological integration as recommended by Wiechman et al. [15] and Patterson et al. [16], and strengthened electrical safety enforcement emphasized by Arnoldo et al. [1] and Dash et al. [11]. Kim et al. [20] similarly reported significantly lower physical component scores in electrical burn amputees at discharge, as discussed above for proximal amputation. Shen and Dai [21] recently advocated for multidisciplinary cooperation and functional reconstruction as the goal of treatment. Consistent with our findings, a case report by Chai et al. [22] highlighted that psychological support was provided to maintain motivation in a quadruple amputee.

While anemia was associated with worse outcomes, these data do not prove causality. Preoperative anemia correction should be investigated in future randomized studies before being recommended as a definitive intervention. None of the proposed management strategies was directly tested in this observational study. The following recommendations are therefore hypothesis-generating, not evidence-based conclusions.

Limitations

Several limitations warrant consideration. First, the sample size of 49 patients, while reflecting consecutive recruitment over one year, is modest and limits the statistical power for detecting smaller effect sizes or performing extensive multivariable adjustments. Second, the single-center design may limit generalizability to other healthcare settings with different rehabilitation resources. Third, the three-month follow-up captures early functional recovery but not long-term adaptation, and the use of both in-person and telephonic outcome assessments, while necessary for geographical access, may have introduced measurement bias despite literature supporting telephonic SIP comparability. Fourth, aside from motivation (ICIDH b130), we did not assess other psychological variables such as depression, anxiety, or social support. References to “psychological integration” in the discussion should be interpreted with this limitation in mind.

Future multicenter prospective studies with larger cohorts are needed to validate our findings and to develop robust predictive models of functional outcome after electrical burn amputation. Given the observational design, these findings identify associations rather than causal effects. Future randomized studies are needed to determine whether correction of anemia or enhancement of motivation improves postamputation functional outcomes.

## Conclusions

Amputation following electrical burn injury results in significant functional disability, primarily determined by amputation level and injury voltage. Beyond these fixed factors, preoperative anemia and psychological state represent modifiable determinants of recovery. Based on these exploratory findings, we hypothesize that addressing preoperative anemia and patient motivation, alongside appropriate surgical care, could potentially improve functional outcomes. However, this hypothesis requires testing in adequately powered interventional trials. Clinically, it is reasonable to consider integrated pathways that include attention to these factors, but our data do not prove their effectiveness.
